# Immune checkpoint inhibitor-induced toxicity: a real-world analysis of the role of BMI

**DOI:** 10.3389/fonc.2025.1659977

**Published:** 2025-11-06

**Authors:** Calogera Claudia Spagnolo, Rosaria M. Ruggeri, Angela Alibrandi, Martina Laganà, Desirèe Speranza, Salvatore Cannavò, Massimiliano Berretta, Mariacarmela Santarpia

**Affiliations:** 1 Department of Biomedical and Dental Sciences and Morphofunctional Imaging (BIOMORF), University of Messina, Messina, Italy; 2 Endocrinology Unit, Department of Human Pathology of Adulthood and Childhood DETEV “G. Barresi”, University of Messina, Messina, Italy; 3 Unit of Statistical and Mathematical Sciences, Department of Economics, University of Messina, Messina, Italy; 4 Department of Chemical, Biological, Pharmaceutical and Environmental Sciences, University of Messina, Messina, Italy; 5 Division of Medical Oncology Unit, “Gaetano Martino” Hospital, Messina, Italy; 6 Department of Clinical and Experimental Medicine, University of Messina, Messina, Italy; 7 Department of Human Pathology of Adulthood and Childhood DETEV “G. Barresi”, University of Messina, Messina, Italy

**Keywords:** immune checkpoint inhibitor, body mass index, immune-related adverse events, obesity, gender

## Abstract

Immune checkpoint inhibitors (ICIs) have radically changed the therapeutic landscape of several cancers. However, only a limited number of predictive factors are currently available in clinical practice to select patients for immunotherapy. The impact of excess weight on ICI toxicity and efficacy is presently under debate. This study was aimed at evaluating the occurrence of immune-related adverse events (irAEs) among cancer patients on ICI therapy according to baseline body mass index (BMI) and gender. The association with clinical outcomes was also analyzed.

**Patients and methods:**

One-hundred thirty patients (93 males, 37 females, median age 67 years) with diverse types of advanced cancer treated with ICIs at a single university hospital were included in the study. Patients with a previously diagnosed thyroid dysfunction were excluded from this analysis.

**Results:**

A number of irAEs occurred in 51 patients (39.2%; 33 males, 18 females). Their development significantly correlated to BMI. Overweight/obese patients experienced a higher (59.5% *vs* 40.5%; p<0.001), and earlier (8 *vs* 10.6 weeks; p=0.003) occurrence of irAEs than normal weight patients. About 65% of overweight/obese patients had an associated dysmetabolic state (i.e., hypertension, glycemic disturbances and/or dyslipidemia) and displayed higher prevalence of irAEs than those without comorbidities (p=0.019). At multivariate regression analyses, BMI was confirmed as an independent predictor of risk for developing AEs (p<0.001), with an odds ratio (OR) of 3.182 for overweight/obese patients. No differences in BMI or gender emerged in progression-free survival (PFS) and overall survival (OS) rates.

**Conclusions:**

irAEs occurred more frequently in overweight/obese patients, mainly with metabolic abnormalities. These data underline the importance of a comprehensive clinical assessment, including weight and dysmetabolic comorbidities, of patients at baseline and during ICI therapy.

## 1 Introduction

Immune checkpoint inhibitors (ICIs) can restore the immune response against cancer by blocking inhibitory molecules, such as cytotoxic T lymphocyte-associated protein 4 (CTLA-4), programmed death-1 (PD-1) and its ligand (PD-L1), expressed on immune and/or tumor cells, the so-called immune checkpoints (1–3). Their use has revolutionized the standard of care of cancer patients, providing therapeutic options for many advanced stage tumors considered otherwise untreatable (4–9). ICI treatment has been associated with excellent response rates and improved survival when administered as either first-line therapy or after other treatments (10–12). Some tumor characteristics, such as the expression of PD-L1 on cancer cells, a T-cell inflamed profile (T-cell infiltration), and the mutational and/or neoantigen burden are currently known to predict response to ICIs, although the identification of predictive biomarker for ICI-based therapy is still challenging (13, 14). In particular, the impact of patient-related factors, like sex, age or BMI, remains to be elucidated, as well as the predictive and/or prognostic role of ICI adverse events, to be considered “on-target” side effects (15).

Indeed, as indications for ICI therapy have expanded and numbers of treated patients have increased, a unique profile of toxicity has emerged, characterized by the occurrence of immune-mediated damage of several tissues and organs (16). These immune-related adverse events (irAEs), autoimmune in etiology, are reported in up to 50% and more of treated patients and can potentially affect all organs. Dermatologic, gastrointestinal, hepatic, and endocrine manifestations are the most frequently reported irAEs, while neurological, cardiac or pulmonary side effects rarely occur (16). In particular, thyroid disorders are among the most common endocrine irAEs, mostly under anti-PD-1/PD-L1 treatment, and include hyperthyroidism, hypothyroidism and destructive thyroiditis (thyrotoxicosis progressing to hypothyroidism) (17–19). Other less common endocrinopathies include hypophysitis, adrenal insufficiency, type 1 diabetes, and hypoparathyroidism (19–21). Although ICI-based therapies are typically well tolerated, the risk of potentially severe irAEs, compromising organ function and/or quality of life, is not negligible and increases with combination regimens (17).

Moreover, some (22, 23) but not all (24) studies have found an association between female sex and occurrence of irAEs.

A growing body of evidence suggests that overweight/obesity may be associated with increased immunotoxicity on the one hand (25) and with improved efficacy of immunotherapy on the other hand (26, 27). The mechanisms behind this unexpected favorable association, the so-called “obesity paradox”, are still not clear (28, 29), and the real impact of overweight on irAE development and efficacy still remains to be further defined. The present study was aimed at evaluating the occurrence of irAEs among cancer patients on ICI therapy according to baseline BMI and gender. Further, we analyzed survival outcome difference in these subgroups of patients.

## 2 Materials and methods

### 2.1 Patients

We performed a retrospective/prospective analysis of patients with different types of cancer, at early or advanced stage of disease, treated with ICIs at the Medical Oncology Unit of the University Hospital “Gaetano Martino” of Messina from January 2020 to December 2024. For each patient, we collected the following data: demographic characteristics (gender and age at the start of ICI therapy), type of cancer [non-small-cell lung carcinoma (NSCLC), melanoma, renal cell carcinoma, and others], type and duration of ICI treatment (nivolumab, pembrolizumab, atezolizumab, ipilimumab, cemiplimab, and durvalumab) weight, BMI, and Eastern Cooperative Oncology Group performance status (ECOG PS) (30). Inclusion criteria were age > 18 years, any type of cancer under ICI treatment, a minimum follow-up duration of 3 months. Exclusion criteria were a previously diagnosed thyroid dysfunction, or evidence of abnormal thyroid function tests at baseline, previous treatment with antithyroid drugs or levothyroxine, or ongoing therapy with corticosteroids or immunosuppressive therapy, and the unavailability of important data from medical records before or after treatment. More in detail, 70 patients were not considered for analysis because of incomplete information, 30 patients were further excluded because were not euthyroid at baseline and/or were already under therapy with L-T4. In total, 130 patients with complete information were included in the study.

The study was carried out in accordance with the World Medical Association’s Declaration of Helsinki. Informed consent from each subject for using anonymized data was obtained.

### 2.2 Methods

Clinical and pathological data for all patients treated with ICIs were collected by consulting medical records. BMI was calculated as body weight (kg) divided by height (m) squared and patients were categorized as being underweight (BMI <18.5), normal weight (BMI 18.5 – 24.9), overweight (BMI 25 – 29.9), or obese (BMI >30) based on cut offs suggested by the World Health Organization [https://www.who.int/news-room/fact-sheets/detail/obesity-and-overweight]. The overweight or obese status were also distinguished based on the presence of a dysmetabolic clinical status, defined as the presence of hypertension, glycemic disturbances (diabetes mellitus, and/or insulin resistance and/or impaired glucose tolerance) and/or dyslipidemia. irAEs were reported and graded according to the National Cancer Institute’s Common Terminology Criteria for Adverse Events (CTCAE), version 5.0 (31).

Treatment efficacy was assessed in terms of overall survival (OS), which was recorded from the beginning of treatment until the observation of death from any cause during follow-up or loss, and in term of progression-free survival (PFS) recorded from the beginning of treatment until the progression of disease, according to the RECIST v 1.1 criteria (32). All biochemistry serum measurements, including hormonal assessment, were performed centrally at the laboratory of the University Hospital of Messina, and were measured both at baseline and at each hospital admission using commercial kits with routine methods.

### 2.3 Statistical analysis

Numerical data are expressed as median and interquartile range and the categorical variables as number and percentage. The examined variables were not normally distributed, as verified by a Kolmogorov–Smirnov test. Consequently, the nonparametric approach was used. In order to compare patients with or without irAE occurrence, the Mann–Whitney test was applied for numerical variables and the Chi Square test (or Likelihood ratio test or exact Fisher test, as appropriate) for categorical variables. Some boxplots were generated to better visualize the differences between two groups of patients. In order to identify possible significant predictors of irAE occurrence (yes or no), logistic regression models were estimated. The explicative power of the following covariates was tested: age, sex, weight, BMI, performance status, type of cancer, type (anti-PD-1, anti-PD-L1, anti-CTLA-4) and duration of ICI treatment, etc. In addition, the predictive power of the interactions between BMI and cancer type, gender, or treatment type was also evaluated. Therefore, a multivariate logistic regression model was estimated inserting only the covariates that were statistically significant at univariate approach [i.e., weight, BMI category, performance status (sec. ECOG) and positive family history of autoimmune disease]. The results were expressed as odds ratio (OR), 95% confidence interval (95%C.I.) and p-value. Kaplan Meier curves were generated to better visualize patient survival time, with reference to OS and PFS, taking into account two stratification factors: AEs and BMI category. The survival analysis was detailed reporting the number of subjects, the number of events, the number and percentage of censored data, the median time with its standard error and its 95% confidence interval, and the Log-Rank test for comparing stratification factors. Statistical analyses were performed using SPSS Statistics for Window v22.0. A p-value < 0.05 was considered to be statistically significant.

## 3 Results

### 3.1 Study cohort

One hundred thirty patients (93 males and 37 females; male/female ratio was 2.51) with complete information were included in the study. Baseline characteristics of the overall cohort are presented in **Table 1**.

**Table 1:** Baseline characteristics of the overall cohort of cancer patients.

	Total patients	Male patients	Female patients	p-value
Number of patients	130	93 (71.5%)	37 (28.5%)	–
Age (years)	67 (range 32-85)	68 (range 32-85)	61.5 (range 38-84)	0.866
Weight (kg)	70.5 (range 48-116)	73 (range 48-116)	64 (range 50-90)	0.267
BMI	22.1 (range 18.1-36.7)	23.5 (range 18.1-36.7)	21 (range 19.1-32.05)	0.285
BMI category				
Underweight	3 (2%)	3 (3.2%)	0	–
Normal weight	83 (63.8%)	55 (59.1%)	28 (75.7%)	0.507
Overweight	37 (28.5%)	30 (32.3%)	7 (18.9%)	0.341
Obese	7 (5.4%)	5 (5.4%)	2 (5.4%)	0.671
Malignancy				
NSCLC	72 (55.4%)	54 (58%)	18 (48%)	0.715
Other types of tumor	58 (44.6%)	39 (41.9%)	19 (52%)	0.672
Melanoma	30 (23.1%)	18 (19.4%)	12 (32.4%)	–
Kidney	10 (7.7%)	6 (6.5%)	4 (10.8%)	–
Head-neck	8 (6.2%)	6 (6.5%)	2 (5.4%)	–
Bladder	7 (5.4%)	7 (8.75%)	0 (0%)	–
Pleural mesothelioma	2 (1.5%)	2 (2.2%)	0	–
Skin	1 (0.8%)	0 (0%)	1 (2.7%)	–
ICI type				
Anti-CTLA-4 Ipilimumab	10 (7.7%)*	8 (10%)*	2 (6.7%)	0.834
Anti-PD-1/PD-L1	120 (92.3%)	72 (90%)	28 (93.3%)	0.943
Pembrolizumab	57 (43.9%)	39 (41.9%)	18 (48.6%)	–
Nivolumab	54 (41.5%)	41 (44.1%)	13 (35.1%)	–
Atezolizumab	6 (4.6%)	4 (4.3%)	2 (5.4%)	–
Durvalumab	2 (1.5%)	1 (1.1%)	1 (2.7%)	–
Cemiplimab	1 (0.8%)	0 (0%)	1 (2.7%)	–
Previous antineoplastic treatment				
Naïve	70 (49.2%)	47 (50.1%)	23 (62.2%)	0.627
Chemotherapy	49 (42.3%)	39 (41.9%)	10 (27%)	0.367
TKI	11 (8.5%)	7 (7.5%)	4 (10.8%)	0.834
Disease status during ICI therapy				
Clinical benefit	56 (43.1%)	38 (40.9%)	18 (48.6%)	0.741
Disease progression	50 (38.5%)	39 (41.9%)	11 (29.7%)	0.490
Death from any cause	24 (18.5%)	16 (17.2%)	8 (21.6%)	0.811

CTLA-4, cytotoxic T lymphocyte-associated antigen-4; PD-1, programmed death receptor 1; PD-L1, programmed death-ligand 1; ICI, immune checkpoint inhibitors; TKI, tyrosine kinase inhibitors; Clinical benefit: including stable disease, partial response and complete response.

*2 patients were treated in combination with nivolumab.

The median age was 67 years (range 32-85). Primary tumors were non-small cell lung carcinoma (NSCLC) (n=72, 55%), melanoma (n=30, 23%), renal cell carcinoma (n=10, 7.7%), and others (n=18, 14.3%). Cancer patients received anti-PD-1 (nivolumab/pembrolizumab/cemiplimab, n= 112, 86%), anti-PD-L1 (atezolizumab/durvalumab, n=8, 6%), anti-CTLA-4 (ipilimumab, alone n=10; or in association with nivolumab, n=2), as first-line (n=63) or subsequent lines (n=60) of therapy (after conventional chemotherapy and/or TKI), or as adjuvant treatment (n=7).

At baseline evaluation, median BMI in the whole cohort was 22 kg/m^2^ (range 18-37); median body weight 70.5 kg (range 48 – 116). According to WHO classification, 3 patients (2.3%) were defined as underweight, 83 patients (63.8%) as having a normal weight, 37 patients (28.5%) as overweight and 7 patients (5.41%) as obese. Overall, 33.9% of patients (n=44) had a BMI ≥25 kg/m2, and 65.9% of them had a history of hypertension, and/or glycemic disturbances and/or dyslipidemia (that is an associated dysmetabolic status).

Thyroid function tests at baseline were within normal limits in all patients, but fifteen (11%) had positive thyroid autoantibodies (TPOAb and/or TgAb) at baseline. None of them was under L-T4 therapy or was taking any drugs interfering with thyroid function.

### 3.2 Incidence and spectrum of adverse events

During treatment, irAEs occurred in 51 patients (39.2%; 33 males and 18 females; median age 69 years), without significant differences between the two sexes (p=0.289). Among them, 41 (78%; 31.5% of the whole cohort) developed thyroid dysfunction (either hypothyroidism or thyrotoxicosis) without difference in sex (p= 0.578). Primary hypothyroidism was the most common irAE, occurring in 39 patients (30% of the whole cohort), including 14 patients who experienced transient thyrotoxicosis with subsequent progression to hypothyroidism, and 25 patients who developed hypothyroidism without any preceding recognized thyrotoxicosis. The 39 patients who developed hypothyroidism received levothyroxine (mean dose of 1.6 μg/kg/day) for the entire duration of ICI treatment. Persistent hyperthyroidism requiring anti-thyroid treatment occurred in two patients. None of the patients who experienced transient thyrotoxicosis was prescribed with glucocorticoids.

Patients who experienced thyroid irAEs showed a higher prevalence of non-thyroidal irAEs (p=0.003). Overall, 29 patients (22.3% of the entire cohort) developed non-thyroid irAEs [cutaneous (n=9), gastro-intestinal (n=9), pulmonary (n=2), rheumatic (n=8), and a single case of adrenalitis], and difference by sex was significant (p=0.007), female patients being more frequently affected than male patients. Among these, 19 patients developed thyroid dysfunction as an irAE, without differences between the two sexes (p=0.08). Overall, irAEs were more frequently recorded in female patients, but thyroid disorders occurred equally in both sexes (**Table 2**). Interestingly, no patient in our cohort developed severe irAEs (grade 3–4 according to the Common Terminology Criteria for Adverse Events) and none had to permanently discontinue ICI treatment. This is partially in line with data from the literature since thyroid irAEs, the most common type of irAEs in our cohort, are usually low grade if promptly diagnosed. However, even with regards to non-endocrinological irAEs, no event of grade >2 and no patient had to permanently discontinue ICI treatment in our series. This unexpected finding may be due to the design of the study, that was first conceived to assess endocrinological irAEs. Hence, we excluded patients with a previously diagnosed thyroid dysfunction that could potentially represents an important predisposing factor for other irAEs. Moreover, only patients with fully documented hormone status at baseline and during follow-up were included, further decreasing the sample size.

**Table 2:** Incidence of irAEs according to gender.

irAEs 51	Total of patients 130	Male patients 93	Female patients 37	p-value
Thyroid irAE	41 (31.5%)	28 (30.1%)	13 (35.1%)	0.578
Hypothyroidism	25 (60%)	16 (57.1%)	9 (69.2%)	*-*
Thyrotoxicosis	16 (40%)*	12 (42.9%)	4 (30.8%)	*-*
Other irAE	29 (22.3%)	15 (16.1%)	14 (37.8%)	0.007
Cutaneous	9 (31.1%)	5 (33.3%)	4 (28.6%)	*-*
Gastrointestinal	9 (31.1%)	5 (33.3%)	4 (28.6%)	*-*
Rheumatological	8 (27.5%)	4 (26.7%)	4 (28.6%)	*-*
Respiratory	2 (6.9%)	1 (6.7%)	1 (7.1%)	*-*
Other endocrine irAE (adrenalitis)	1 (3.4%)	0	1 (7.1%)	*-*
Thyroid irAE + Other irAE	19	10 (10.8%)	9 (24.3%)	0.080

*14 patients experienced a transient thyrotoxicosis with a subsequent progression to hypothyroidism and two patients developed persistent hyperthyroidism.

### 3.3 Predictors of irAEs and time of onset

Patients who developed irAEs under ICI treatment had similar age and gender distribution. However, despite no differences by gender emerging between patients experiencing thyroid dysfunction (p=0.578), non-thyroidal irAEs occurred more frequently in female than male patients and the difference was significant (p=0.007).

**Figure 1:**
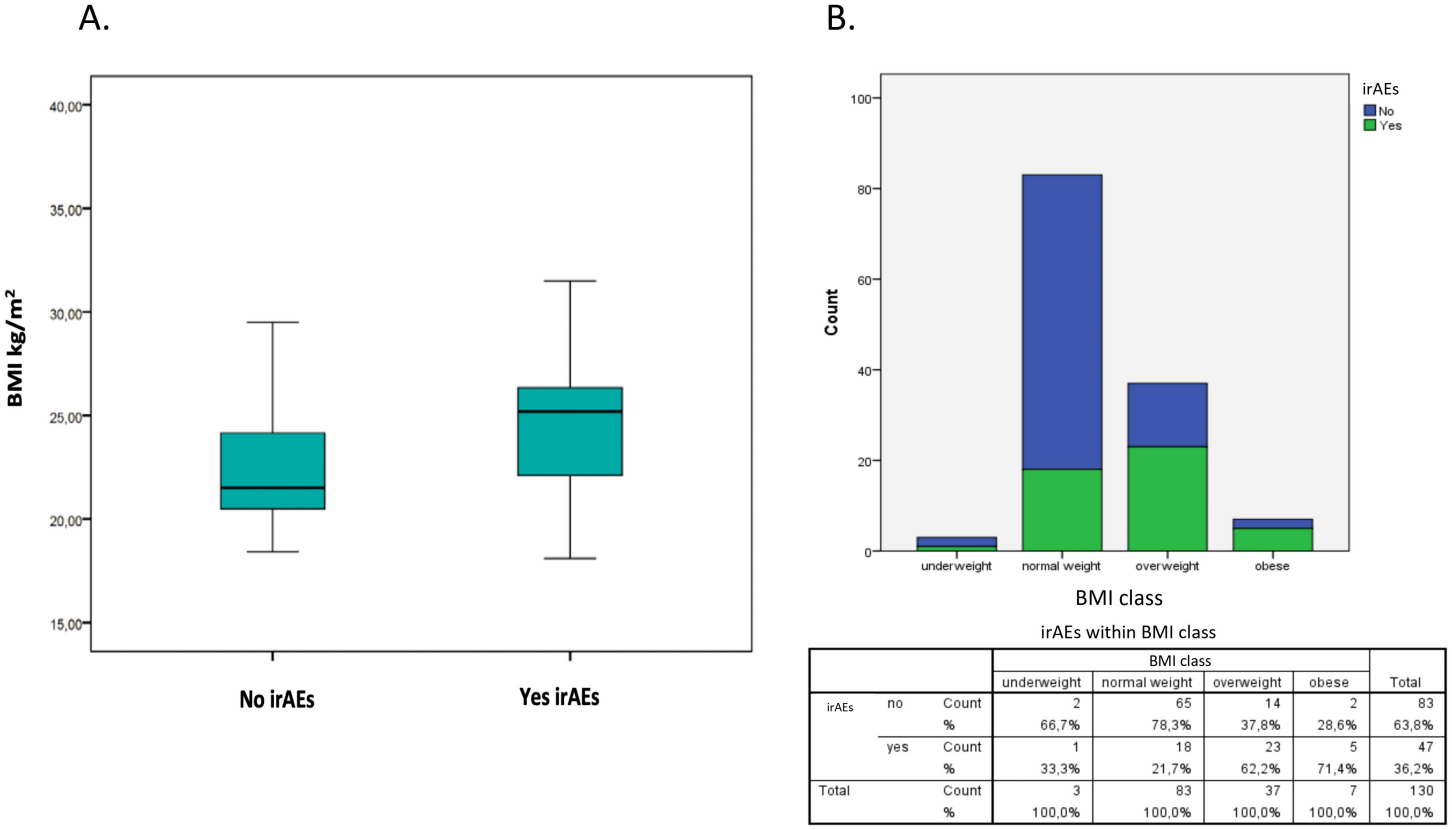
Development of irAEs and BMI. **(A)** The boxplot on the left shows the distribution of patients who did not develop irAEs (no) based on BMI, while on the right it shows the distribution of those who developed irAEs (yes). Development of irAEs was associated with higher BMI. The prevalence of irAEs was 59.5% in overweight/obese patients vs 40.5% in normal weight patients (p<0.001). **(B)** The graphic shows the prevalence of irAEs according to BMI category.

**Figure 2:**
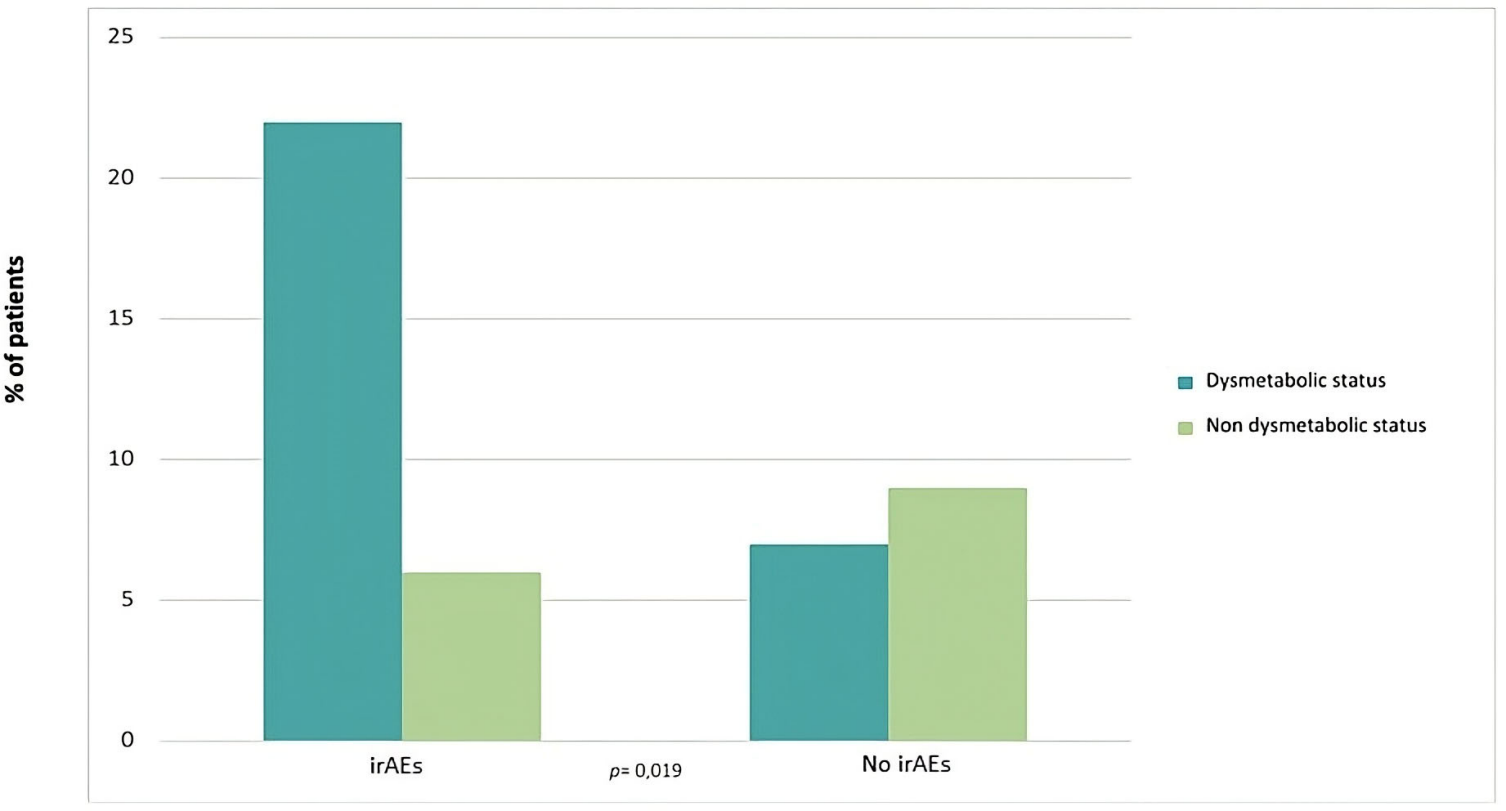
Prevalence of irAEs in dysmetabolic overweight/obese patients. The graphic shows the prevalence of irAEs according to the presence of a dysmetabolic state in overweight/obese patients. Dysmetabolic overweight/obese patients had a higher prevalence of irAEs compared to those who did not have these associated comorbidities (22/29 vs 6/15; p = 0.019).

Development of irAEs was associated with higher BMI (**Figures 1A, B**). The prevalence of irAEs was 59.5% in overweight/obese patients vs 40.5% in normal weight patients (p<0.001). Patients who developed irAEs had higher body weight (75.5 ± 12 kg vs 70.2 ± 11 kg, p = 0.017) and higher BMI (25 ± 3.5 kg/m^2^ vs 22.7 ± 3 kg/m^2^, p = 0.002) than patients who did not, in both sexes. The prevalence of irAEs was higher among overweight/obese patients compared to normal weight patients, whether they are considered (59.5% vs 40.5%; p<0.0001) or divided by sex (males, 67% vs 33%; p=0.001; females, 57% vs 43%, p=0.011). About 65% of overweight/obese patients had an associated dysmetabolic state, defined as the presence of hypertension and/or glycemic disturbances (insulin resistance, diabetes, impaired glucose tolerance) and/or dyslipidemia (n=29/44). These dysmetabolic overweight/obese patients had a higher prevalence of irAEs compared to those who did not have these associated comorbidities (22/29 vs 6/15; p=0.019) (**Figure 2**).

At uni- and multivariate regression analyses, BMI, more in detail BMI category, was confirmed as an independent predictor of risk for developing irAEs (p<0.001), with overweight/obese patients having an OR of 3.182 compared to normal weight/underweight patients. Higher BMI and a better ECOG performance status were associated with the occurrence of irAEs (p<0.001, and p=0.013, respectively). Also, a positive family history of any autoimmune disease was a predictor of risk (p< 0.001) (**Table 3**).

**Table 3:** Results of univariate and multivariate logistic regression models for the occurrence of irAEs.

Variable	Univariate			Multivariate		
	Crude OR	95% CI	*p*-value	Adjusted OR	95% CI	*p*-value
Gender	1.413	0.636 – 3.140	0.396	–	–	–
Age	1.016	0.979- 1.054	0.393	–	–	–
Weight	**1.043**	**1.008-1.080**	**0.020**	0.984	0.928-1.044	0.594
BMI category	**3.182**	**1.678-6.031**	**0.000**	**3.538**	**1.501- 8.340**	**0.004**
Tumor type						
*Lung vs other* *Melanoma vs other*	0.845 1.575	0.337-2.116 0.549-4.521	0.719 0.399	**-** **-**	**-** **-**	**-** **-**
Performance status (ECOG)	**2.937**	**1.254-6.879**	**0.013**	**3.486**	**1.019- 11.924**	**0.047**
Positive family history of autoimmune disease	**11.953**	**29.544-40.836**	**0.001**	**6.864**	**2.240-21.034**	**0.001**
BMI category * gender	1.022	0.989-1.057	0.195	**-**	**-**	**-**
BMI category * tumor type	1.011	0.993-1.030	0.223	**-**	**-**	**-**
BMI category * treatment type	1.020	0.999-1.042	0.167	**-**	**-**	**-**

CI: confidence interval; OR: odds ratio.

Significant values ​​in bold.

The median time from first treatment with ICI to the development of any irAE was 8 weeks (range 1–60 weeks), and about 60% of irAEs occurred within the first 9 weeks. Thyroid irAEs usually preceded or coincided with the occurrence of non-endocrine irAEs, being the first side effects reported in almost all patients in our cohort. When subdividing our patients according to gender, median time to first appearance of irAEs was 6 weeks (range 2–12 weeks) in females and 8 weeks (range 1–60 weeks) in males, with female patients experiencing an earlier onset of irAEs than males (p = 0.047). When stratifying time to first appearance of irAEs by BMI category, the median time to develop any irAE was 7 weeks (mean 8.5 ± 6.5 weeks, range 3–60 weeks) in overweight/obese patients compared to 8 (mean 10.7 ± 12.7 weeks, range 3-30) in normal weight/underweight patients, so that irAEs occurred earlier in patients with higher BMI (p=0.003). Thus, overall, overweight/obese patients experienced irAEs more frequently and earlier than normal weight/underweight patients.

**Figure 3:**
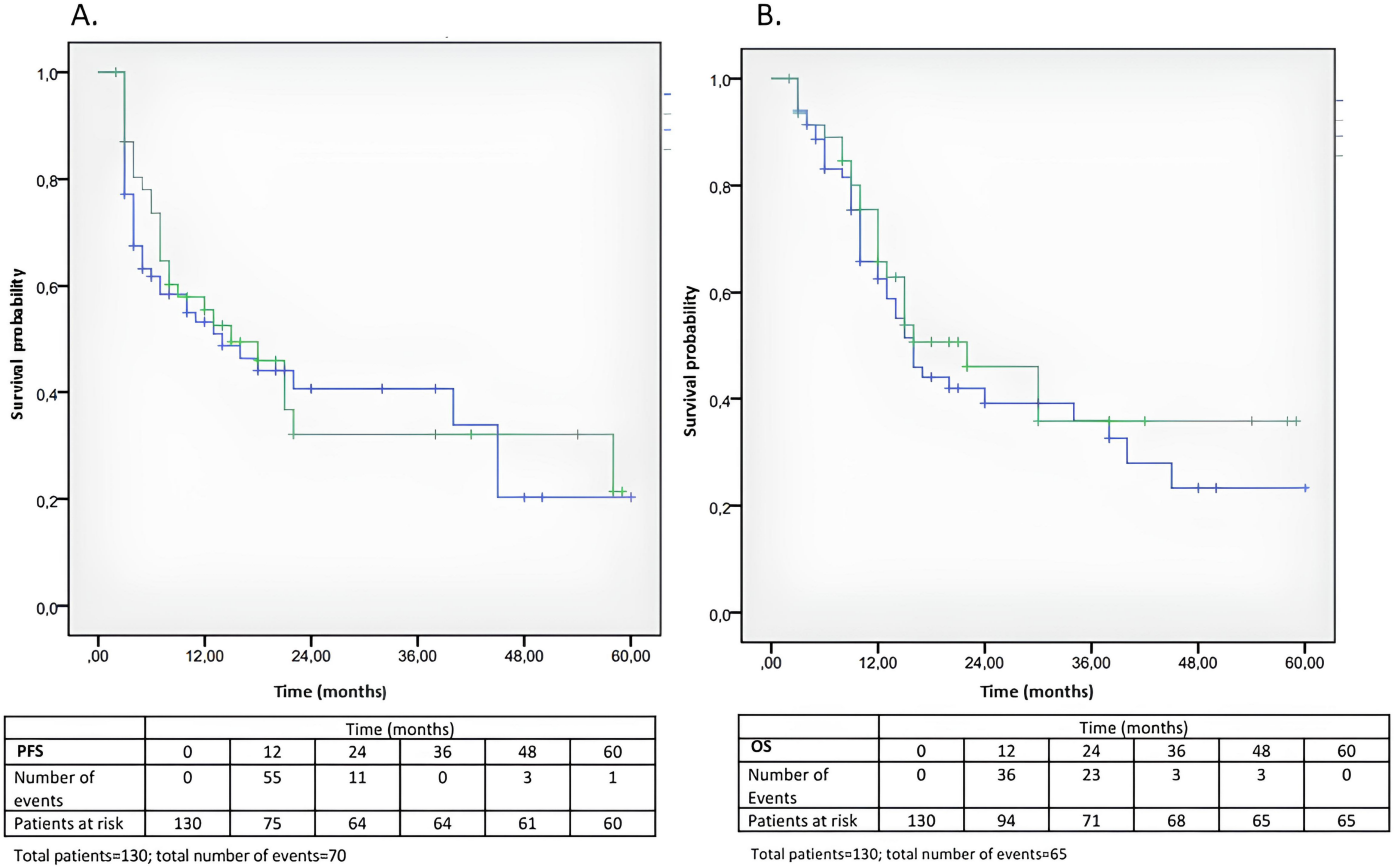
**(A, B)** Kaplan-Meier analysis of PFS and OS according to the development of irAEs. **(A)** Kaplan-Meier curves of PFS in the group of patients that developed irAEs (green) and in those who did not develop irAEs (blue) (median PFS 15 months vs. 14 months, p = 0.662) **(B)** Kaplan-Meier curves of OS in the group of patients that developed irAEs (green) and in those who did not develop irAEs (blue) (median OS 22 months vs. 16 months, p = 0.446) (see **Tables 4A**, **B**).

**Figure 4:**
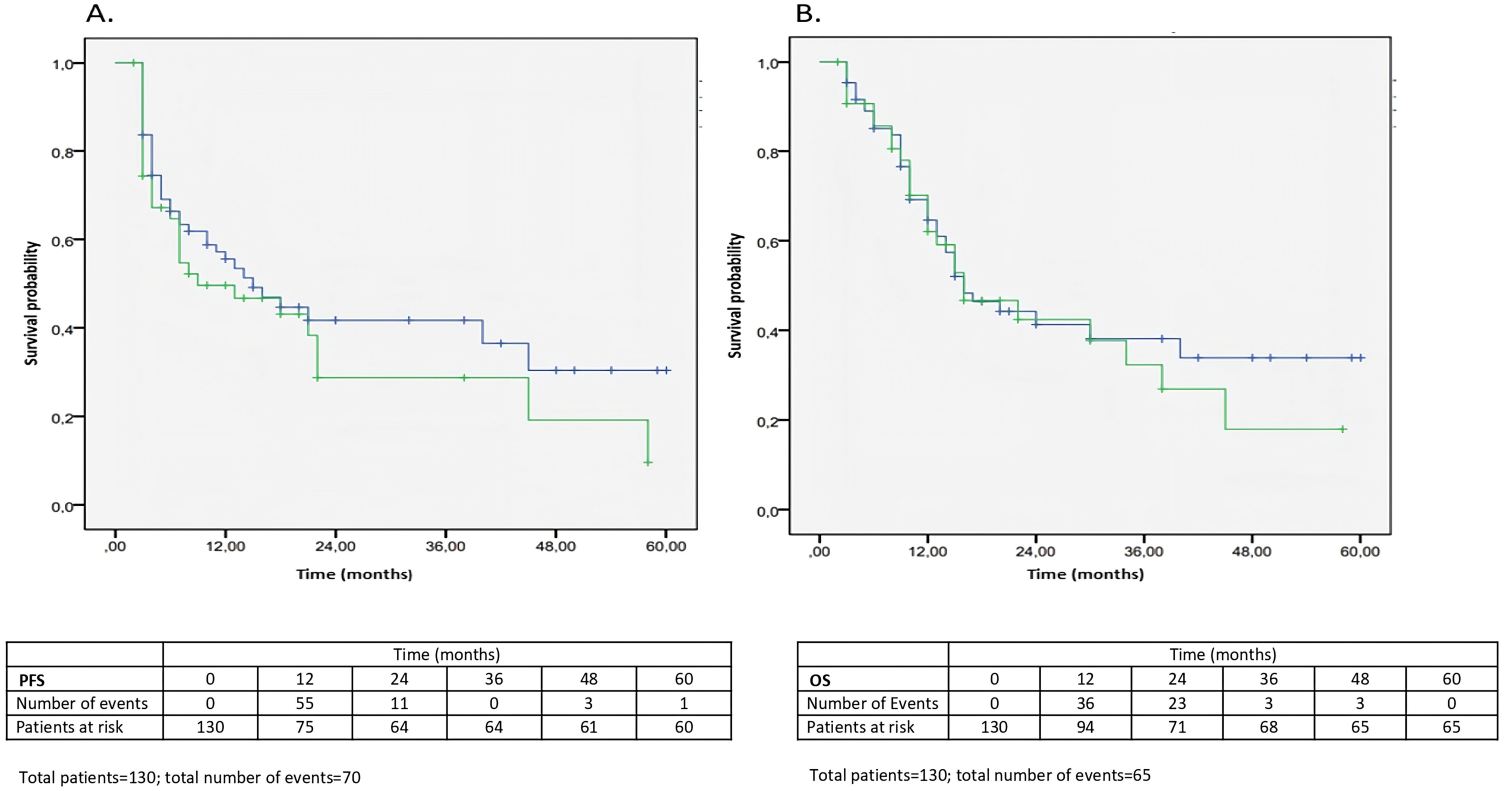
**(A, B)** Kaplan-Meier analysis of PFS and OS according to BMI category (overweight/obese or normal weight/underweight). **(A)** Kaplan-Meier curves of PFS in the group of overweight/obese patients (green) and normal weight/underweight patients (blue) (median PFS 9 months vs. 15 months, p = 0.313). **(B)** Kaplan-Meier curves of OS in the group of overweight/obese patients (green) and normal weight/underweight patients (blue) (median OS 16 months vs. 16 months, p = 0.629) (see **Tables 4C**, **D**).

Overall, no significant statistical differences in PFS and OS emerged. The median PFS and OS were, respectively, 14 and 16 months. Neither PFS nor OS were significantly different both between the group of patients that developed irAEs compared to those who did not develop irAEs (median PFS 15 months *vs*. 14 months, p = 0.662; median OS 22 months *vs*. 16 months, p = 0.446) (**Figures 3A, B**) (**Tables 4A**, **B**), and between the group of overweight/obese patients compared to normal weight/underweight patients (median PFS 9 months *vs*. 15 months, p = 0.313; median OS 16 months *vs.* 16 months, p = 0.629) (**Figures 4A, B****) (****Tables 4C**, **D**). However, it is worth noting the 6-month advantage in OS in the group of patients that developed irAEs, as well as a longer, although not statistically significant, PFS in the normal weight/underweight patient group.

**Table 4A:** Kaplan-Meier curves for OS (factor: AEs).

AEs	N of subjects	N. of events	Censored		
			N	%	
No	83	42	41	49.4%	
Yes	47	23	24	51.1%	
Total	130	65	65	50.0%	
	Median				
	Estimation	Standard error	95%Confidence interval		
			Lower limit		Upper limit
No	16.0	1.749	12.571		19.429
Yes	22.0	5.411	11.395		32.605
Total	16.0	2.753	10.605		21.395
Comparison between factors			Chi-square		p-value
Log Rank (Mantel-Cox)			0.580		0.446

**Table 4B:** Kaplan-Meier curves for PFS (factor: AEs).

AEs	N of subjects	N. of events	Censored	
			N	%
No	83	43	40	48.2%
Yes	47	27	20	42.6%
Total	130	70	60	46.2%
	Median			
	Estimation	Standard error	95%Confidence interval	
			Lower limit	Upper limit
No	14.0	4.805	4.582	23.418
Yes	15.0	3.728	7.692	22.308
Total	14.0	3.449	7.241	20.759
Comparison between factors			Chi-square	p-value
Log Rank (Mantel-Cox)			0.191	0.662

**Table 4C:** Kaplan-Meier curves for OS (factor: BMI).

BMI	N of subjects	N. of events	Censored	
			N	%
Normal weight/underweight	86	40	46	53.5%
Overweight/obese	44	25	19	43.2%
Total	130	65	65	50.0%
	Median			
	Estimation	Standard error	95%Confidence interval	
			Lower limit	Lower limit
Normal weight/underweight	16.0	2.879	10.356	21.644
Overweight/obese	16.0	4.507	7.166	24.834
Total	16.0	2.753	10.605	21.395
Comparison between factors		Chi-square	p-value	
Log Rank (Mantel-Cox)		0.233	0.629	

**Table 4D:** Kaplan-Meier curves for PFS (factor: BMI).

BMI	N of subjects	N. of events	Censored	
			N	%
Normal weight/underweight	86	42	44	51.2%
Overweight/obese	44	28	16	36.4%
Total	130	70	60	46.2%
	Median			
	Estimation	Standard error	95%Confidence interval	
			Lower limit	Lower limit
Normal weight/underweight	15.0	3.423	8.291	21.709
Overweight/obese	9.0	4.338	0.498	17.502
Total	14.0	3.449	7.241	20.759
Comparison between factors		Chi-square		p-value
Log Rank (Mantel-Cox)		1.019		0.313

## 4 Discussion

The present study investigated the impact of gender and BMI on irAE development and efficacy in a cohort of patients with different types of cancer. In our single center cohort, 51 patients (39.2%) developed an irAE, thyroid dysfunction being the most common one, consistent with previously published real-world data (33–36).

With regard to gender differences, overall, irAEs were more frequently observed in female patients, with the relevant exception of thyroid disorders that occurred equally in both genders. Unlike other studies including a large number of patients with lung cancer (37–39), we found a female prevalence in irAEs, as reported in other series (23). Moreover, female patients experienced an earlier onset of irAEs than males.

Regarding the impact of BMI, we found that it was strongly associated with the occurrence of irAEs. Indeed, the prevalence of irAEs was significantly higher among overweight/obese patients compared to normal weight patients in both sexes, and patients with higher BMI were at increased risk of developing an irAE, with an OR of 3.182 compared to normal weight/underweight patients.

Evidence regarding the association between BMI and irAEs among cancer patients receiving ICIs is limited, and sometimes conflicting. Some studies reported a significantly higher incidence of irAEs in patients with higher BMI (25, 35, 39), while other studies failed to demonstrate such an association. Two meta-analyses explored the association between BMI and irAEs among patients with cancer receiving ICIs, and both concluded that high BMI was associated with a higher rate of irAEs (40, 41). Our study provides further evidence in support of a positive correlation between BMI and development of irAEs, reporting a 3-fold increase of the risk of irAEs among overweight/obese patients. Moreover, irAEs occurred earlier in overweight/obese patients than in normal weight/underweight patients, although without any difference in severity.

The mechanisms of such an intriguing association between BMI, a surrogate measure of body fat, and ICI therapies are not completely understood. Obesity is a low-grade inflammatory metabolic condition that has been associated with both cancer and autoimmunity (42, 43). Indeed, obesity is associated with increased adipose tissue, metabolic disturbances (hyperglycemia), higher levels of insulin, and insulin-like growth factors (IGFs), with potent mitogenic activity (44–46). Moreover, it has been associated with increased secretion by adipocytes of pro-inflammatory cytokines (*TNF*-α, IL-6 and IL-1β) and adipokines (leptin, adiponectin, resistin), which could affect T cell function, resulting in Th1/Th2 imbalance and promoting a pro-inflammatory state (47, 48). Such a pro-inflammatory condition is well known to predispose to the occurrence of autoimmune disorders (49, 50); hence, excess body weight may promote the development of irAEs. Moreover, fat accumulation leads to enhanced infiltration of pro-inflammatory CD8^+^ T cells into adipose tissue, accompanied by a reduction in adipose-resident regulatory T cells (Tregs) (51). Additionally, other obesity related factors such as dietary habits, genetic susceptibility, and microbiome may contribute to increased occurrence of irAEs in obese patients.

Worthy of note, our study is the first to suggest that irAEs occur more frequently in overweight/obese patients with a concurrent dysmetabolic state, defined as the presence of hypertension and/or glycemic disturbances and/or dyslipidemia, suggesting that low grade meta-inflammation, well known to be associated with obesity and related metabolic disorders, may represent a predisposing condition for development of irAEs in patients with higher BMI. Therefore, baseline BMI and related dysmetabolic conditions should be considered among the potential risk factors for the development of irAEs, along with other potential predictors, such as a family or personal history of autoimmune disorders, the use of immunotherapeutic combinations or previous TKI treatment. This would help clinicians in identifying patients who are at higher risk for irAEs, thereby personalizing therapeutic choices and clinical monitoring.

Alternatively, overexposure to treatment may occur in overweight patients due to an increased dose calculation based on mass weight (52). In this light, sarcopenic obesity, a not rare condition in oncologic patients, represents a complex and emerging factor in cancer patients undergoing ICI therapy. Characterized by the coexistence of low muscle mass and excess adiposity, it has been associated with increased toxicity and poorer clinical outcomes in various cancer settings (53–55). Recently, a systematic review and meta-analysis examining the impact of sarcopenia on cancer patients treated with ICIs found that sarcopenia is associated with an increased risk of irAEs, though the relationship with irAEs was less clear (56). Moreover, sarcopenic obesity may act as a confounding factor, influencing both the incidence of irAEs and treatment efficacy, complicating the interpretation of clinical outcomes (53). This dual role underscores the importance of considering body composition, beyond simple measures of body weight, when interpreting clinical associations. Further studies are needed to elucidate its precise impact and underlying mechanisms.

Even more complex is the relationship between obesity, cancer outcomes and response to cancer treatment in the context of ICI treatment. Obesity has been recognized as a risk factor and a negative prognostic factor for several cancers, worsening oncological outcomes, including recurrences, disease-free survival, all-cause and cancer specific mortality (29). Nevertheless, evidence suggests that overweight and obesity may be associated with better oncological outcomes than normal weight, and an inverse relationship between BMI and mortality (the so-called ‘obesity paradox’) has been found in several cancers at advanced stage, although the underlying mechanisms are not fully understood (29, 50).

A possible suggested mechanism could be the better objective responses to immunotherapy observed in obese compared to non-obese patients, with significantly longer PFS and OS (57). A high BMI has been associated with better clinical outcomes to ICI therapy. Complex interactions between the adipose tissue and tumor cells have been described (58). The modulation of the tumor microenvironment by obesity-associated molecules, including hormones and pro-inflammatory cytokines (e.g. TNF-α, IL-6) can play a key role in promoting tumor development and progression and, at the same time, in enhancing T-cells function and immune responses to ICIs. Besides inflammation, other mechanisms can modulate the effects of obesity in cancer patients, including alterations of insulin-like growth factor pathways, induction of hypoxia and HIF-1α signaling, and modulation of microbiota (59). The role of BMI as a predictor of toxicity from anti-neoplastic drugs should be further explored. In advanced cancer patients treated with ICIs, several studies reported better outcomes, in terms of longer PFS and/or OS, in overweight/obese patients compared to patients with normal BMI, with differences by sex (26, 27, 60, 61).

In our cohort, we failed to find any relationship between obesity, occurrence of irAEs and treatment efficacy in terms of either PFS and/or OS. However, it is widely known that survival outcomes can be influenced by several factors related to patients and/or cancer. We hypothesize that this may be due to several factors. First, the sample size of the study was limited, and our patient cohort included various tumor types and stages — although the majority were advanced — which can independently be associated with different prognoses, regardless of treatment. Moreover, since this was a real-world, observational study, patients with some significant comorbidities (i.e., cardiovascular) and with ECOG PS 2, who are usually excluded from large registration trials, were also included. Finally, other important factors could have potentially affected survival outcomes, including previous therapeutic lines and the great variability among patients in the timing and methods used for disease response evaluation, as for clinical practice. However, we decided to assess PFS and OS because these outcomes can better reflect the long-term efficacy of immunotherapy compared to treatment response.

Some limitations should also be noted, including: (i.) the retrospective design; (ii.) the relatively limited number of patients; (iii.) the inclusion of patients with different types of cancer, introducing some clinical heterogeneity and potential biases. Interestingly, our cohort of 130 patients had no grade 3/4 irAEs or irAEs leading to treatment discontinuation, which is lower than would be expected according to literature data (25, 27). In the multi-center retrospective study by Cortellini and coworkers, including 1070 advanced cancer patients treated with PD-1/PD-L1 inhibitors, higher BMI was significantly related to higher occurrence of G3/G4 irAEs and therapy discontinuation (25). The absence of high-grade irAEs in our series may be due to the small sample size compared to larger studies, and/or to recruitment bias. Indeed, our study was first designed to assess endocrinological AEs, hence we excluded patients with a previously diagnosed thyroid dysfunction that could potentially represents an important predisposing factor for other irAEs. Endocrinological AEs, the most common type of irAEs, are usually low grade, and, if promptly diagnosed and treated in the context of an experienced team of endocrinologists and oncologists, do not worse and/or lead to treatment discontinuation. Overall, this should be acknowledged as a limitation of our work, preventing us from conducting a more in-depth investigation into the relationship between BMI and the severity of irAEs, as demonstrated in other studies.

Overall, major strengths of the study are: (i.) access to complete information (hospital-based data) regarding patients at baseline and during ICI-treatment; (ii.) a real-life scenario, that assesses irAEs presentation and management in regular clinical practice, thereby reflecting real adherence to treatment/intervention and outcomes; (iii.) a homogeneous cohort of patients belonging to the same geographical area followed-up at a single center. Thus, this real-life study provides evidence on how treatments perform in routine clinical practice, capturing a broader and more heterogeneous patient population than controlled trials. It offers valuable insights into effectiveness, safety, and feasibility in everyday care, complementing existing literature. We have to acknowledge that the small number of patients limits subgroup analyses and precludes drawing definitive conclusions, while noting that the observed trend of higher irAE rates in overweight/obese patients with metabolic comorbidities remains an interesting finding. Further studies on larger series are needed to verify whether these results can be extrapolated to other populations and confirmed on large series.

## 5 Conclusions

In conclusion, despite advances in the knowledge of the peculiar profile of toxicity of ICI-based therapies, many questions regarding irAEs remain to be fully addressed. These include the role of predisposing factors like BMI and gender, as well as the possible association between occurrence of irAEs and response to ICI treatment. Also, clinical and biochemical predictors of the risk for developing irAEs are needed. In our well-characterized cohort of patients treated with ICIs, we confirmed that overweight/obesity is associated with increased risk of irAEs, with a notable predictive value, mostly when accompanied by dysmetabolic conditions. However, no clear association between BMI and immunotherapy efficacy was observed, in terms of either PFS or OS. These results may help oncologists to identify the patients who are most likely to develop irAEs, improving the management of their patients in a real-life scenario.

## Data Availability

The raw data supporting the conclusions of this article will be made available by the authors, without undue reservation.
